# Epiphytic and endophytic colonisation of tomato plants by the entomopathogenic fungus *Beauveria bassiana* strain GHA

**DOI:** 10.1080/21501203.2019.1707723

**Published:** 2020-01-05

**Authors:** Oumi Nishi, Hirotoshi Sushida, Yumiko Higashi, Yuichiro Iida

**Affiliations:** National Agriculture and Food Research Organization, Tsu-city, Japan

**Keywords:** *Beauveria bassiana*, epiphyte, endophyte, microcycle conidiation, biocontrol agent

## Abstract

*Beauveria bassiana*, known for its entomopathogenic characteristics, is the most widely used biocontrol agent against many insect pests and may also be active against soil-borne pathogens. It inhabits the surfaces or inner tissues of various plant species without causing any visible signs or symptoms. Here we show that *B. bassiana* strain GHA, the active ingredient of a commercial microbial insecticide, colonises tomato plants. GHA grew on intact leaf surfaces of tomato in high humidity, but never entered stomata. Viable hyphae and conidia were detected, and the population on inoculated leaves significantly increased until 14 days after inoculation. On tomato leaves, GHA conidiated normally via conidiophores and phialides, and also via microcycle conidiation (conidiophores and phialides form directly from germ tubes and produce conidia). Hyphae were also detected inside the rachis, even more frequently after plant surfaces were scarified. These results suggested that *B. bassiana* strain GHA can grow epiphytically and endophytically on tomato plants.

## Introduction

Mitosporic hypocrean fungi such as *Beauveria bassiana* (Bals.-Criv.) Vuill. (Hypocreales: Cordycipitaceae), *Akanthomyces* spp. Lebert (previously known as *Lecanicillium* spp. W.Gams and Zare) (Hypocreales: Cordycipitaceae) and *Metarhizium anisopliae* (Metsch.) Sorok. (Hypocreales: Clavicipitaceae) are known entomopathogens and have been developed as biocontrol agents against a wide range of insect pests (Vega et al. 2009). Because they can protect plants from infection by nematodes and pathogens (Shinya et al. 2008; Vega et al. 2008, 2009; Bamisile et al. 2018), they play multiple roles in integrated pest management (IPM) strategies and sustainable crop production. Epi- and endophytic entomopathogenic fungi also provide efficient biocontrol of serious leaf-inhabiting pests such as thrips, whitefly, and leaf-miner fly, that have developed resistance to chemical pesticides (Kliot et al. 2016). In fact, endophytic entomopathogens provide especially biocontrol effect against leaf-miner moths (Klieber and Reineke 2016; Barta 2018).

In addition to the wide use of *B. bassiana* as a microbial insecticide of agricultural pests (Faria and Wraight 2007), certain strains have been reported to suppress soil-borne fungal diseases in several plants. Seed treatment with strain 11–98 showed that it colonised cotton and tomato seedlings and suppressed disease caused by *Rhizoctonia solani* (Ownley et al. 2004, 2008). Take-all disease of wheat caused by *Gaeumannomyces graminis* var. *tritici* and basal rot of onion caused by *Fusarium oxysporum* f. sp. *cepae* were inhibited by pretreatment with *B. bassiana* (Renwick et al. 1991; Flori and Roberti 1993). *B. bassiana* was also antagonistic against the oomycete *Pythium myriotylum* and nematodes *Meloidogyne marylandi, M. incognita*, and *Globodera pallida* (Bamisile et al. 2018). Antifungal secondary metabolites produced by *B. bassiana* EABb 09/16-Su were possibly contribute to suppressions of fungal pathogens of olive (Lozano-Tovar et al. 2013). Thus, *B. bassiana* apparently has wide plant compatibility and is a good candidate for controlling numerous plant pathogens. *B. bassiana* has also been found as natural epiphytes, endophytes, and rhizosphere colonisers (Meyling and Eilenberg 2006; Behie et al. 2015; Garrido-Jurado et al. 2015). Some isolates of this species were established as such plant associates, although in some case the establishments were transient (Posada and Vega 2005; Quesada-Moraga et al. 2014; Klieber and Reineke 2016; Resquín-Romero et al. 2016; Garrido-Jurado et al. 2017; Jaber and Ownley 2018).

When the commercial bioinsecticide Botanigard® was used to control insect pests, we found that powdery mildew of tomato did not appear in the greenhouses. Thus, *B. bassiana* strain GHA, the active ingredient of Botanigard®, might act as a dual biocontrol agent to suppress both insect pests and pathogens of tomato. *B. bassiana* strain GHA has a wide spectrum of pathogenicity against insects and has been used in commercial bioinsecticide products to control many pests of crops (Ugine et al. 2005; Lohmeyer and Miller 2006; Faria and Wraight 2007; Portilla et al. 2019), however, little is known about its epi- and endophytic abilities with regard to tomato plants.

The common approach in the use of the biocontrol agents is to spray spore suspensions directly onto plant leaves or stems, which results in a temporarily high concentration of fungal spores on plant surfaces. To enhance and extend the beneficial effects of the agent, understanding the events and processes of phyllosphere colonisation by *B. bassiana* strain GHA should help in developing practical applications of Botanigard®. In this study, we assessed the epi- and endophytic abilities of the strain GHA on and in tomato plants.

## Materials and methods

### Fungal isolates and transformation

*B. bassiana* strain GHA isolated from a commercially available bioinsecticide Botanigard® ES (Arysta LifeScience, Tokyo, Japan) and its transformants were used throughout the study. Strain GHA was transformed with the vector pAL1gpd including the GFP gene, placed downstream of a glyceraldehyde-3-phosphate dehydrogenase promoter cloned from *Aspergillus oryzae* (GenBank accession AAIH02000003) and bar (glufosinate resistant) gene, using protoplasts (Shimizu and Kurisu 1987) and polyethylene glycol (Ying and Feng 2006). The vector was constructed from pAL1 (Lichius et al. 2012) obtained from the Fungal Genetics Stock Centre (www.fgsc.net). Single-spored cultures of transformants were observed with a confocal laser scanning microscope (CLSM) (LSM700; Zeiss, Oberkochen, Germany), and one of the transformants with appropriate GFP fluorescence was selected for further study. Monosporic cultures of the wild-type and GFP-transformed strain (GHAgfp) were grown on sabouraud dextrose yeast extract agar (SDYA; glucose 20 g, peptone 2 g, yeast extract 2 g, agar, 15 g per L) at 25°C for 2 week and stored at 4°C.

*B. bassiana* strains were grown on SDYA plates for 14 d at 25°C. Conidial suspensions were collected in sterile distilled water containing 0.05% v/v Tween 20 and filtered through sterile cheesecloth to remove hyphae. The suspensions were washed twice by centrifugation for 5 min at 2500 × *g*. Concentrations of conidia were determined using a haemocytometer.

### Observation of epiphytic growth

Seedlings of tomato *Solanum lycopersicum* cv. Regina (Sakata Seed, Kanagawa, Japan) were grown in pots and incubated at 25°C and a 16 h light/8 h dark photoperiod. Aboveground parts of 6–8-week-old tomato plants were sprayed with a conidial suspension of GHAgfp (1 × 10^7^ conidia/ml; ca. 0.5 ml per leaf), then placed in a moist chamber at 60% or 100% humidity. Plants treated with the same solution served as the control. Sprayed leaves, petioles, and stems were fixed at 3 and 7 days post inoculation (dpi) in 2.5% v/v glutaraldehyde in 0.1 M sodium cacodylate buffer (pH 7.2) for more than 24 h at 4°C for scanning electron microscopy (SEM) using a JEOL JSM-35 SEM (JEOL, Tokyo, Japan) as previously reported (Iida et al. 2018). The samples were dehydrated using a graded series of ethanol (50–100%), then immersed in 100% *tert*-butanol. Specimens were coated with gold–palladium (20:80) in a Polaron E5100 sputter coating unit (Polaron Equipment, Hertfordshire, UK). Photographs were taken with a JEOL JSM-35 SEM (JEOL Co., Ltd., Tokyo, Japan) at 20 kV.

Colonisation of GHAgfp on tomato leaves was also observed using the CLSM at 1, 3, and 7, and 14 dpi. Images were acquired by excitation with a 488 nm laser and 495–515 nm filter to detect fluorescence emitted by the transformant and edited using LSM software ZEN 2010 (Zeiss).

### Detection of ghagfp from tomato leaf surfaces

Four 8-week-old tomato plants were sprayed with a conidial suspension of GHAgfp (1 × 10^5^ conidia/ml; ca. 0.5 ml per leaf). Three squares (1 × 1 cm) were trimmed from each plant and immersed in a microtube containing 1 ml of sterile distilled water plus 0.05% v/v Tween 20 at 0, 7 and 14 dpi. The tubes were vigorously vortexed for 5 min, then a dilution series was prepared and spread on selective SDYA (SDYA amended with glufosinate ammonium 0.2 g and chloramphenicol 0.1 g/l). Glufosinate ammonium was added to the medium to detect only the transformant with glufosinate resistant gene. Population numbers (colony forming units: CFUs) of GHAgfp on the leaf surfaces were estimated after 2 weeks at 25°C. The experiment was repeated three times. The data for the three replicates at each time point were compared using the Wilcoxon rank sum test with *p*-value adjusted by Holm’s method (*H*_0_: There is no difference in population numbers among the three time points) in R version 3.4.3 (www.r-project.org/).

### Detection of ghagfp from internal tomato tissue

One microlitre of a conidial suspension of GHAgfp (1 × 10^5^ conidia/ml) was dropped on the rachises of 8-week-old tomato plants. Rachises were also wounded by pricking with a sterile needle or scratching with a sterile toothpick, then 1 µl of a conidial suspension of GHAgfp (1 × 10^5^ conidia/ml) was dropped on each wound (Supplementary Figure 1). The inoculated plants were placed in a moist chamber at 100% humidity for the first 7 days, then the lid was opened (40–60% RH). Fourteen rachises, with one inoculation site on each, were excised from the plants at 14 dpi, then surface-sterilised in 70% v/v ethanol for 2 min and 2 min in 2.1–3.4% w/v sodium hypochlorite, and then washed twice with sterile distilled water. Surface-sterilised rachises were cut into three segments (ca. 0.5 cm) with a razor blade; one with the inoculation site at the centre, and segments on each side of the inoculated segment (Supplementary Figure 1). *B. bassiana* that grew from the rachis segments was detected on selective SDYA plates after 2 weeks. The experiment was repeated three times. Data were compared using Fisher’s exact test with *p*-value adjustment by Benjamini and Hochberg’s method (*H*_0_: There is no difference in detection rates among the three treatments) in R version 3.4.3.

Four inoculated rachises, with wounds on each, were also excised from the plants at 7 and 28 dpi and hand-cut into round slices with a razor blade. *B. bassiana* GHAgfp in rachises was detected using the CLSM. For better detection of *B. bassiana* GHAgfp in wounded parts of the plants, samples were fixed in formalin (37% formaldehyde, 8% methanol, v/v), bleached with ClearSee (FUJIFILM Wako Pure Chemical, Osaka, Japan) according to the manufacturer’s protocol, and stained with FITC-conjugated lectin from *Triticum vulgaris* (wheat) (Merck, Darmstadt, Germany).

## Results and discussion

### Epiphytic growth and microcycle conidiation

**Figure 1.**
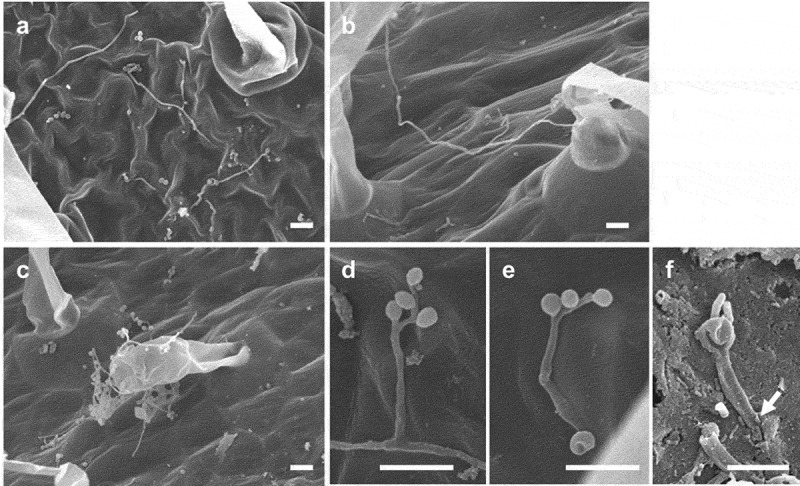
Growth of *Beauveria bassiana* GHAgfp on tomato surfaces. Hyphae on a leaf (a), petiole (b), and stem (c) (7 dpi). Conidiophore and phialides that formed from hypha (d) and conidia on phialides that differentiated from a germ tube (e) on a leaf (7 dpi). An arrow shows the penetration site on a scratched wound on a rachis (f) (3 dpi). Bars are 10 µm (a-e) and 5 µm (f)

Epiphytic growth of *B. bassiana* GHAgfp on tomato leaves was observed by SEM and CLSM. Since GHAgfp was not detected at 3 dpi in low humidity (60%), we used the fungus at high humidity (100%). At 7 dpi, conidia had germinated, and hyphae were elongating on the leaves, petioles and stems; phialides were also conidiating (Figure 1(a–d)). Hyphae randomly elongated on the surfaces and were never seen in stomata. In addition, conidia germinated, and the germ tubes directly produced a conidiophore and phialides, which formed two or three conidia (Figure 1(e)). This phenomenon, termed microcycle conidiation, is well known in fungi (Hanlin 1994; Jung et al. 2014).

Some of the conidia produced on the cuticle were generated on phialides, which in turn were produced directly on germ tubes. Such microcycle conidiation, observed in more than 100 fungal species across various taxonomic groups (Hanlin 1994), is generally induced by heat or starvation stresses and considered a survival strategy in unfavourable environments. For example, *in vitro* starvation for carbon and nitrogen sources induces microcycle conidiation by a strain *B. bassiana* (Bosch and Yantorno 1999). Thus, this phenomenon suggests that tomato surfaces are unfavourable for vegetative growth of *B. bassiana* GHA, presumably because carbon and nitrogen sources are scarce. Barta (2018) reported that a *B. bassiana* strain on horse-chestnut leaves formed an enlargement (perhaps the initiation of a microcycle conidium) at the tip of its germ tubes. It is unknown whether secondary conidia produced via microcycle conidiation possess insecticidal properties.

**Figure 2.**
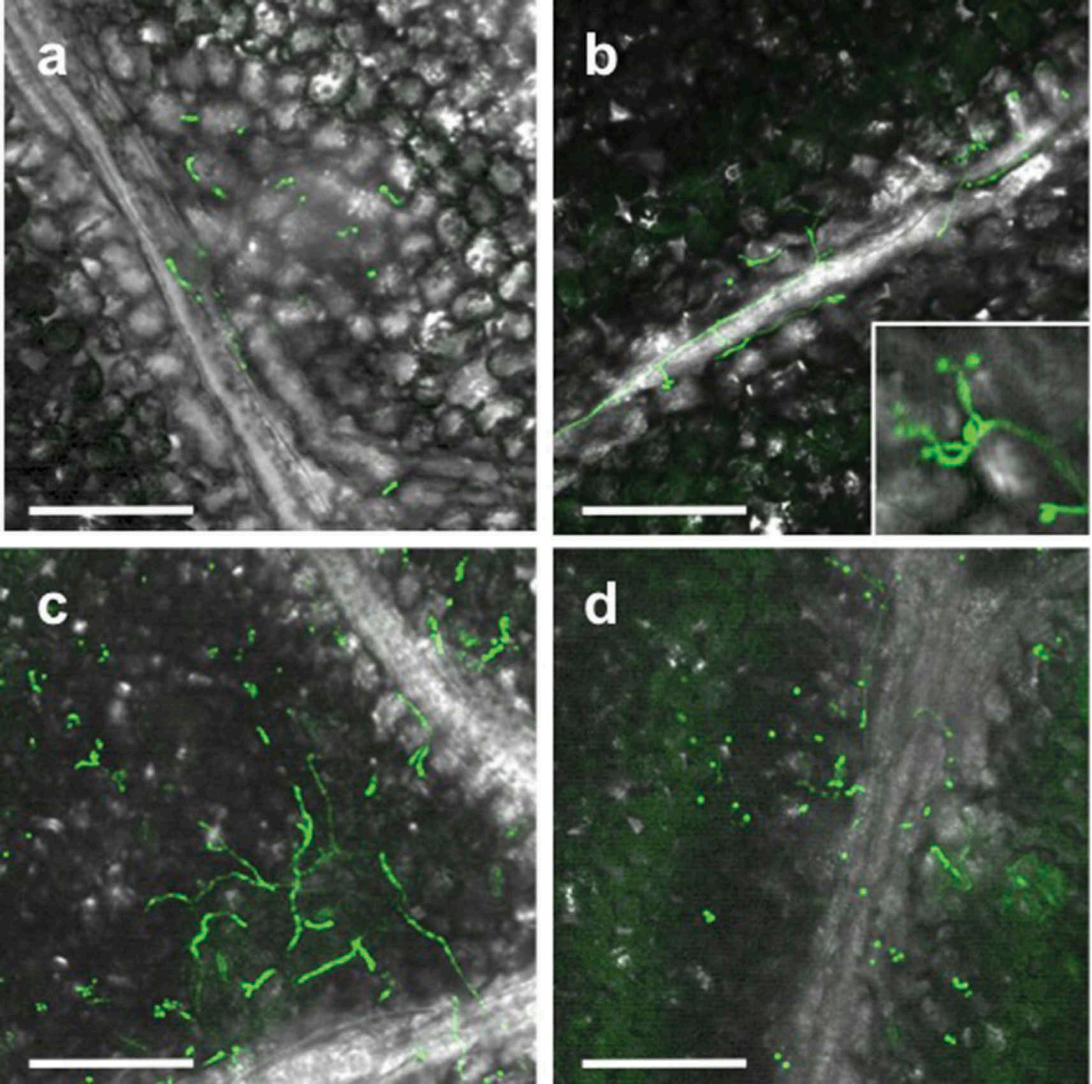
Viable conidia and hyphae of *Beauveria bassiana* strain GHAgfp on tomato leaf at 1 (a), 3 (b), 7 (c) and 14 (d) dpi. Enlarged conidia and phialides are shown in b. Bars are 100 µm

**Figure 3.**
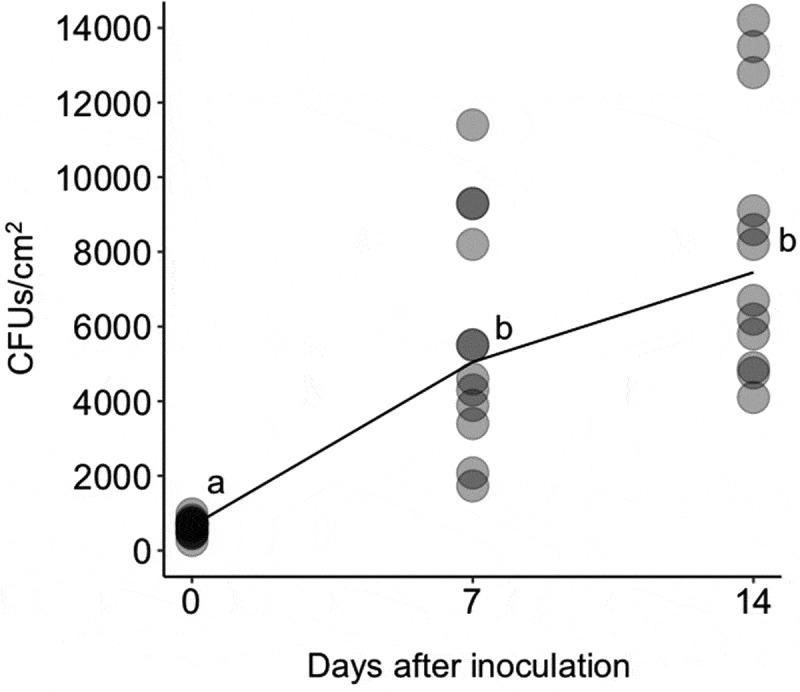
Colony forming units (CFUs) of *Beauveria bassiana* GHAgfp on tomato leaves at 0, 7 and 14 dpi. Different letters indicate a significant difference in CFUs (median) among times (*p* < 0.005, Wilcoxon’s rank sum test with Holm’s *p*-value adjustment)

Since strain GHAgfp expresses GFP under the control of the constitutive promoter, only viable cells emitted GFP fluorescence. On leaves, conidia germinated by 1 dpi (Figure 2(a)), and hyphal elongation and conidiation were observed by CLSM at 3 dpi (Figure 2(b)). GFP fluorescence from hyphae and conidia was also detected until 14 dpi (Figure 2(c,d)). Similarly, population numbers of *B. bassiana* on inoculated leaves until 14 dpi significantly increased (Figure 3). These results indicated that *B. bassiana* GHA can grow epiphytically on tomato surfaces.

Epiphytic growth with conidial production was also reported for an entomopathogenic and mycoparasitic fungus *Verticillium lecanii* on cucumber plants and the timing of the conidiation was possibly associated with its biocontrol effect against cucumber powdery mildew (Verhaar 1997). The epiphytic nature of *B. bassiana* GHA observed in this study may also contribute to its biological control efficacies against plant pathogens as well as epiphytic pests.

### Endophytic establishment after wounding treatment

**Figure 4.**
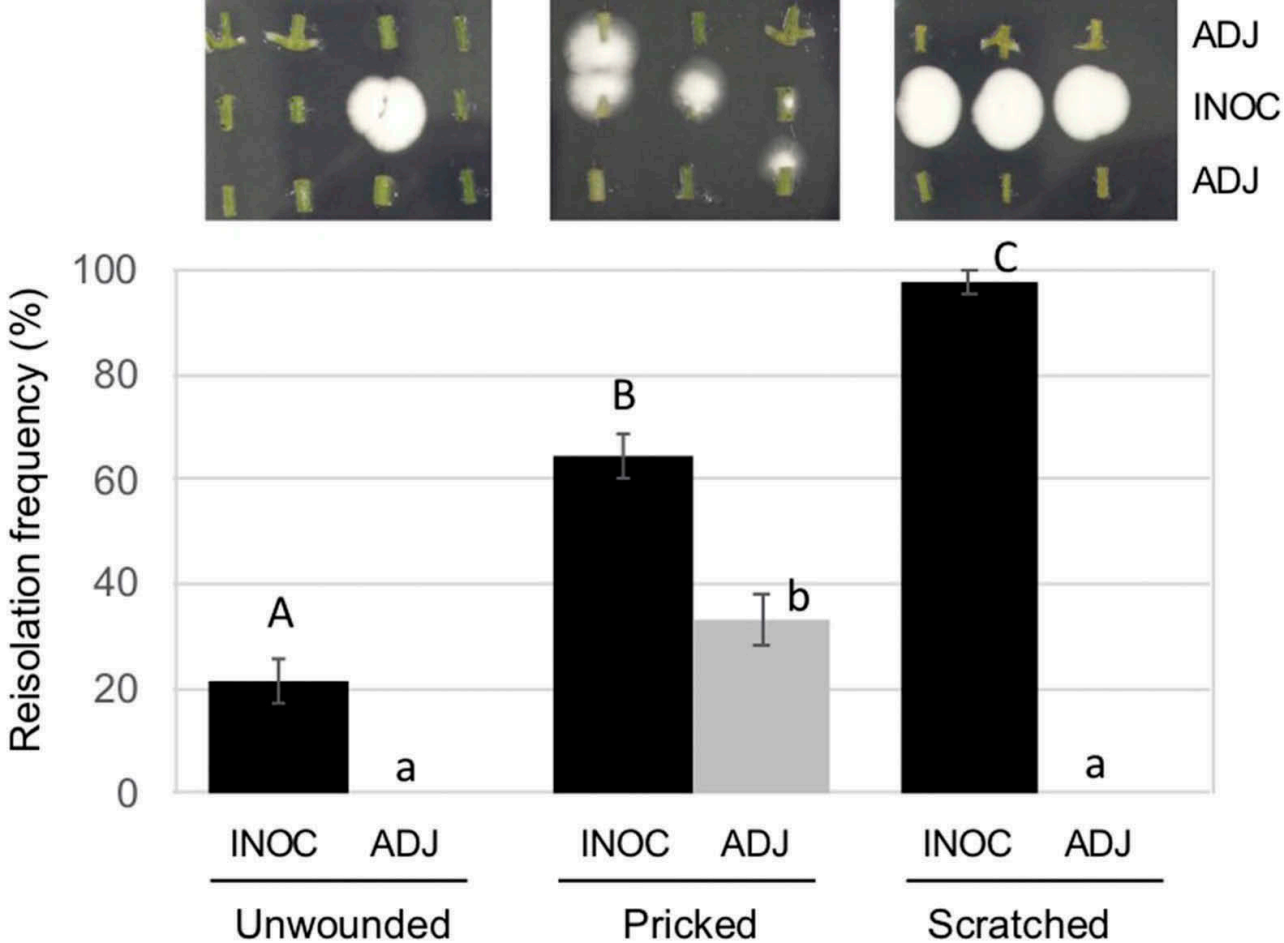
Frequency of reisolation of *Beauveria bassiana* GHAgfp from inoculated rachis segments after wounding and inoculation with a conidial suspension. Inoculated and surface-sterilised segments were cut into three pieces and placed on SDYA containing glufosinate ammonium. Detail procedures are shown in Supplementary Figure 1. GHAgfp was detected from surface-sterilised segments (top row). INOC: segments with inoculation site; ADJ, segments adjacent to inoculated segment. Different letters indicate a significant difference (*p* < 0.05) (upper case: segments with inoculation site, lower case: segments adjacent to inoculated segment) among treatments in Fisher’s exact test with *p*-value adjustment by Benjamini and Hochberg’s method

**Figure 5.**
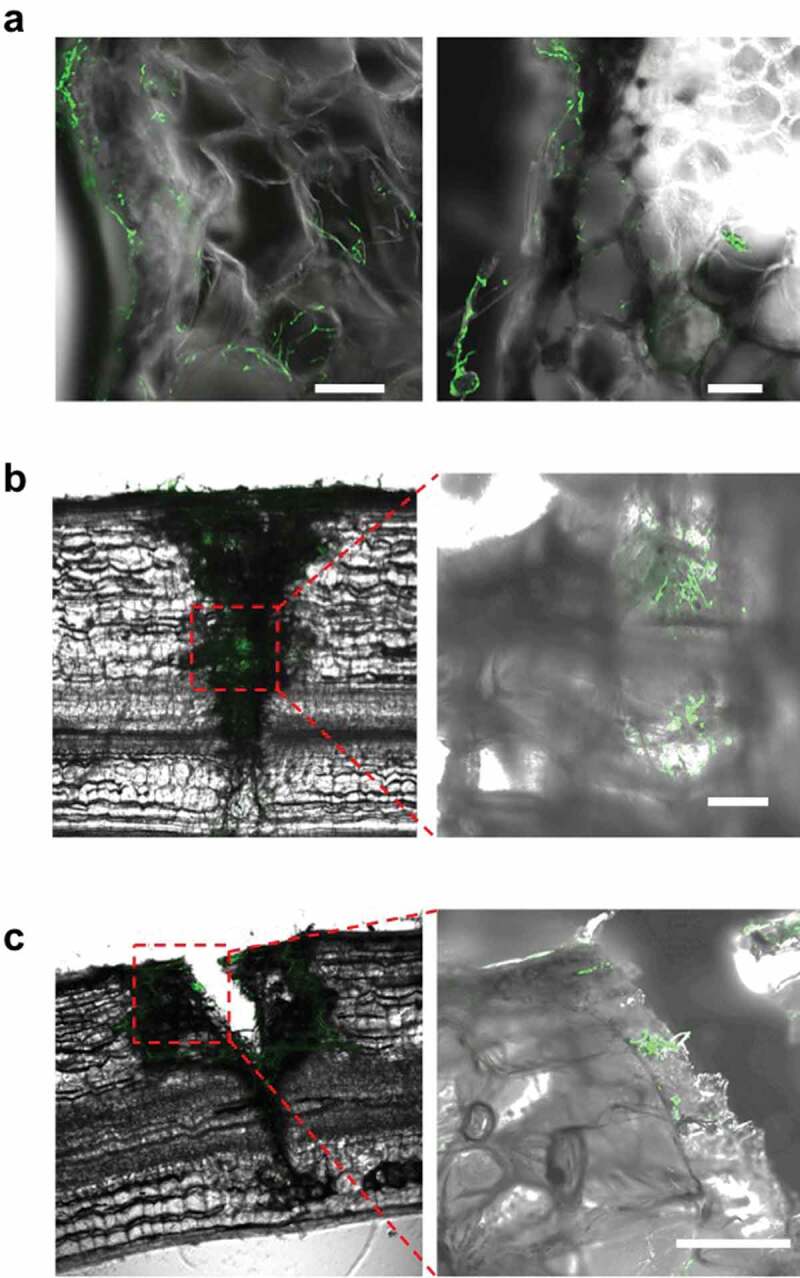
Viable *Beauveria bassiana* GHAgfp in scratch and prick wounds of tomato rachis. (a) Hyphal growth around parenchyma cells under the scratched epidermis at 7 dpi. Viable hyphae in a prick wound at 7 (b) and 28 dpi (c). Area within the red dotted lines is shown at higher magnification in image to the right. Scale bars are 100 µm

*B. bassiana* GHAgfp was reisolated from within tomato rachises with and without wounding (Supplementary Figure 1). The reisolation frequency of GHAgfp from unwounded rachises was very low, about 20% from inoculated segments and 0% from segments adjacent to inoculated segments (Figure 4). GHAgfp was detected from segments with the inoculation site, irrespective of the type of wounding. However, the frequency of detection differed significantly among the treatments: GHAgfp grew from almost all scratched rachises, from about 60% of the pricked rachises and from 20% of the unwounded rachises (Figure 4). GHAgfp hyphae frequently grew through the scratch wounds (Figure 1(f)), but was not reisolated from segments adjoining the inoculated segments unless the rachises had been pricked (Figure 4). In scratch and prick wounds, elongated hyphae of GHAgfp were detected at 7 and 28 dpi (Figure 5), and viable hyphae were visible around parenchyma cells under the scratch wounds. These results suggested that *B. bassiana* GHAgfp penetrates and colonises plant tissues and that colonisation is more frequent after plant surfaces have been wounded. *B. bassiana* GHA infrequently invaded into plant tissues by penetrating cuticles or other natural openings on intact rachises. However, according to Koch et al. (2018), it was also possible that some inoculated conidia were placed directly in stomata and they escaped surface sterilisation.

When applied to various crops to protect against insect pests, *B. bassiana* has been found to grow epiphytically and endophytically (Vega et al. 2008, 2009; Jaber and Ownley 2018). However, the epiphytic and endophytic modes differ depending on the *B. bassiana* strain and host plant species: for example, *B. bassiana* strain ARSEF3113 directly penetrates the intact epidermis of opium poppy and corn leaves without differentiating infection structures such as an appressorium (Wagner and Lewis 2000; Quesada-Moraga et al. 2006), but strain EABb04/01-Tip enters horse-chestnut leaves through stomata (Barta 2018). Strain ATCC74040 germinates and forms hyphae on the surface of various plant species, and it conidiates on the wounded surface of faba beans (Koch et al. 2018). In the present study, strain GHA grew on tomato surfaces, conidiated, and grew intracellularly in the parenchyma, more frequently when the tomato surface was scratched. Its hyphae apparently never entered stomata, penetrated intact cuticles, or developed an appressorium-like structure, even though *B. bassiana* and other entomopathogenic fungi produce such structures on insect cuticles (St. Leger et al. 1991; Kumar et al. 1999; Askary and Yarmand 2007). Whether growth of *B. bassiana* strains is epi- or endophytic growth on plants probably depends on the combination of *B. bassiana* strain and host species.

In the reisolation experiment to assess endophytic colonisation after scratch wounding, *B. bassiana* was detected from nearly all the inoculated segments but never from the adjacent segments, suggesting that GHA lacks the potential to spread *in planta* even though it can penetrate the epidermis through scarified cuticles. Hyphal growth was observed in pricked wounds, but the hypha had not grown into the internal tissue by 28 dpi. These results indicated that the endophytic potential of *B. bassiana* GHA in pricked wounds is low. However, GFP fluorescence of *B. bassiana* GHAgfp in wounds of rachises was detected throughout the experiment (28 dpi), suggesting that this strain can live for a long period in the tomato tissues.

The results of the microscopic observations and the reisolation experiment suggested that scratch wounds facilitate endophytic colonisation of *B. bassiana* GHA in the tomato rachis. *B. bassiana* GHA can grow through the scratched surfaces and reach the internal tissue. During typical cultivation, tomato plants are often wounded by herbivores, bud picking, and accidental physical contact during routine management, which would thus promote endophytic colonisation in cultivation systems using a *B. bassiana* GHA formulation. The endophytic colonisation was not systemic but may contribute to better biocontrol effects because even transient and not-systemic endophytic establishment by spray application contributed to suppressing both sucking and chewing pests (Garrido-Jurado et al. 2015; Resquín-Romero et al. 2016).

## Conclusions

This study has revealed that *B. bassiana* GHA grew epiphytically on intact tomato surfaces in high humidity. GHA also established endophytically near inoculation sites, even more frequently after plant surfaces were scarified. Our report on conidiation by *B. bassiana* on intact plant surfaces is the first, despite numerous studies of *B. bassiana* on various plants, suggesting that the types of growth of *B. bassiana* in association with a plant depend on the specific fungal strain and plant taxon. Thus, for developing effective biocontrol strategies using *B. bassiana*, the growth of the respective strains on the specific plants needs to be evaluated in association with the biocontrol efficacy.
